# Translation into modern standard Arabic, cross-cultural adaptation and psychometric properties’ evaluation of the Lower Extremity Functional Scale (LEFS) in Arabic-speaking athletes with Anterior Cruciate Ligament (ACL) injury

**DOI:** 10.1371/journal.pone.0217791

**Published:** 2019-06-10

**Authors:** Vasileios Korakakis, Michael Saretsky, Rodney Whiteley, Matthew C. Azzopardi, Jasenko Klauznicer, Abdallah Itani, Omar Al Sayrafi, Giannis Giakas, Nikolaos Malliaropoulos

**Affiliations:** 1 Aspetar, Orthopaedic and Sports Medicine Hospital, Doha, Qatar; 2 Faculty of Physical Education and Sport Science, University of Thessaly, Trikala, Greece; 3 Hellenic Orthopaedic Manipulative Therapy Diploma (HOMTD), Athens, Greece; 4 Sports and Exercise Medicine Clinic, Thessaloniki, Greece; 5 National Track & Field Centre, Sports Medicine Clinic, Thessaloniki, Greece; 6 European Sports Care, London, United Kingdom; University of Texas at San Antonio, UNITED STATES

## Abstract

**Background:**

The Lower Extremity Functional Scale evaluates the functional status of patients that have lower extremity conditions of musculoskeletal origin. Regional Arabic dialects often create barriers to clear communication and comparative research. We aimed to cross-culturally adapt the Lower Extremity Functional Scale in modern standard Arabic that is widely used and understood in the Middle East and North Africa region, and assess its psychometric properties.

**Methods:**

Cross-cultural adaptation followed a combination of recommended guidelines. For psychometric evaluation 150 patients with anterior cruciate ligament injury and 65 asymptomatic individuals were recruited. All measurement properties as indicated by the Consensus-based Standards for the selection of health status Measurement Instruments recommendations were evaluated, including content-relevance analysis, structural validity, longitudinal reproducibility, anchor- and distribution-based methods of responsiveness, as well as the longitudinal pattern of change of Lower Extremity Functional Scale in anterior cruciate ligament injured patients’ functional status.

**Results:**

The questionnaire presented excellent internal consistency (α = 0.96), reliability (0.80–0.98), and good convergent validity (ρ = 0.85). For reproducibility testing: minimal detectable change was 9.26 points; for responsiveness assessment: minimal clinically important difference was 9 points and presented moderate effect sizes (Glass’*Δ* = 0.71, Cohen’s *d* = 0.81). Its unidimensionality was not confirmed and an exploratory factor analysis indicated a 2-factor solution explaining 78.1% of the variance.

**Conclusion:**

The Arabic Lower Extremity Functional Scale presented acceptable psychometric properties comparable to the original version. The Arabic version of Lower Extremity Functional Scale can be used in research and clinical practice to assess the functional status of Arabic-patients suffering an anterior cruciate ligament injury.

## Introduction

The Lower Extremity Functional Scale (LEFS) is a patient-reported outcome measure which assesses the functional status of patients who have lower extremity conditions of musculoskeletal origin. The 20-item questionnaire inquires as to the degree of difficulty of performance of each of these activities on a 5-point Likert scale and a score of 80 represents an individual with normal function. [1]

Despite that the number of available patient-reported outcome measures has increased dramatically over the past decades, most of these instruments are developed for English-speaking patients. These tools in order to be used in different language and culture populations require a specific methodology with aim the adequate linguistic translation, but more importantly the cultural adaptation to maintain the content validity of the instrument across different cultures. [2] The LEFS utilizes lay terminology in simple English sentence format making it feasible to translate into modern standard Arabic for a lay population. Regional Arabic dialects often create barriers to clear communication whereas modern standard Arabic is widely used and understood in the Middle East and North Africa region (MENA). Recently, an Arabic version of LEFS [3] was developed, but it was only tested in Saudi Arabian participants. The authors suggested formal testing of the measurement properties of the tool before administration in patients from different Arabic countries. [3] We tested the questionnaire in our institution and we noticed that the translation was literally acceptable but confusing when applied to different Arabic-speaking ethnicities or to patients with low educational level. Specifically, we observed that the researchers maintained culturally irrelevant items and terminology not usually used in Arabic countries or not easily comprehensible by lay population. Accordingly, we decided to develop a version of the questionnaire maintaining its content validity and being understood across most of the Arabic speaking world.

LEFS has been validated in several musculoskeletal conditions and its context-dependent measurement properties have been recently evaluated and summarized. [4] However, the LEFS has been used infrequently in studies examining patients following Anterior Cruciate Ligament (ACL) reconstruction and its psychometric properties have not been determined in this population. [4, 5] Therefore, the main objectives of this study were: i) to translate and cross-culturally adapt the LEFS for a wide spectrum of Arabic-speaking patients and ii) to evaluate its psychometric properties in a cohort with an ACL injury.

## Methods

The translation and cross-cultural adaptation process adhered to published guidelines. [2, 6, 7] The validation of LEFS followed quality criteria on the evaluation of health status questionnaires [8] and the Consensus-based Standards for the selection of health status Measurement Instruments (COSMIN) recommendations. [9]

This study was conducted at the rehabilitation department of our institution. Ethics approval was obtained from the Institutional Review Board (Anti-Doping Lab Qatar—SCH-ADL-A-071), all participants gave written informed consent, and the study was conducted from 2013 to 2016.

### Translation and cross-cultural adaptation

The original LEFS [1] developed in English language and was translated into modern standard Arabic. To ensure uniformity between source and target version special attention was paid to semantic, idiomatic, experiential, and conceptual equivalence. [2] The process followed 8 steps merged from published recommendations [2, 6, 7] (Table 1). Subsequently, proposed psychometric properties [8, 9] to ensure quality of the evaluation of the Arabic LEFS (LEFS-MSAr) were established (S1 File).

**Table 1:** The process of translation and cross-cultural adaptation of the LEFS questionnaire for Arabic-speaking patients.

Steps	Procedures
Step 1: *Initial Translation*	Two bilingual and bicultural translators, whose native language was Arabic, independently produced 2 translations and 2 written reports. One translator (informed) had medical background (physiotherapist) and was aware of the construct of the scale, while the other translator (naïve) had no clinical background (language teacher) but was knowledgeable about the cultural and linguistic nuances of the Arabic language.
Step 2: *Reconciliation* *Committee*	The pair of translators in collaboration with a bilingual committee (3 physiotherapists and a sports medicine physician), a coordinator (researcher with several years of experience in scales development and validation), and a recording bilingual researcher synthesized the 2 translations and through a consensus process harmonized and produced a common initial translation and a written report documenting the synthesis process.
Step 3: *Back Translation*	Two bicultural translators, whose native language was English and who were fluent in the target language, produced 2 independent back translations of the initial questionnaire. Both were uninformed of the concepts explored to avoid information bias, had no medical background, and were blind to the original questionnaire.
Step 4: *Back translation review committee and harmonization*	An independent committee with aim the conceptual equivalence of translation consisting of the translators, members of the research team, and included bilingual clinicians knowledgeable about the content area convened, reached consensus, and developed the pre-final version of the LEFS-MSAr for translation validation. During this process the committee assessed the original questionnaire [1] and each translation (reconciled and back) together with the corresponding written report. Special attention was given by the committee to ensure intertranslation validity and to achieve semantic, idiomatic, experiential, and conceptual equivalence between the source and target questionnaire. [2, 6, 7] Furthermore, the committee discussed “comparability of language” which refers to the formal similarity of words and sentences between the original and back-translated questionnaire, as well as “similarity of interpretability” which refers to the degree to which the versions produce the same response even if the wording differs. [2, 6, 7]
Step 5: *Validation of translation*	The validation of the LEFS-MSAr regarding the success of the translation process was assessed in two ways: a) Formal evaluation of comparability of language and similarity of interpretability by using 7-point Likert scales ranging from 1 (extremely comparable/extremely similar) to 7 (not at all comparable/not at all similar). [6] Six bilingual individuals (3 men and 3 women, mean age 30.8 years and range 26 to 35 years) rated each original and back-translated item. Following this process each mean score >3 (comparability) and >2.5 (similarity) requires review for possible correction. b) Cognitive debriefing: the LEFS-MSAr was tested for cognitive equivalence [7] by 10 native Arabic-speaking patients at various educational levels representing the Gulf region population (Qatar, UAE, Jordan, Syria, Lebanon, Morocco, Tunisia, and Egypt) in order to capture the differences in Arabic dialects across the Middle East/North Africa region (mean age_(range)_ 30.6_(19–45)_ years). The same 10 individuals were also used for the item-content relevance analysis (see content validity).
Step 6: *Review and finalization*	Considering the comments from the former process the committee made all necessary modifications for improvement.
Step 7: *Proofreading*	A proofreading company checked the final version for spelling, diacritical, grammatical, or other errors.
Step 8: *Pretesting*	The pre-final version of the LEFS-MSAr was administered to 20 Arabic-speaking patients suffering from lower limb musculoskeletal conditions (14 men and 6 women, with age_(range)_ of 36.6_(19–59)_ years). Following the completion of the questionnaire, each individual was formally interviewed regarding the comprehension of items and the chosen response as part of the assessment of face and content validity. Upon completion of pre-testing a committee convened and the pre-final version without corrections was accepted as the final version of the LEFS-MSAr questionnaire (S1 File).

### Sample size calculation and participants

The sample size required for the study was based on the intraclass correlation coefficient (ICC) and the maximum width of the 95% confidence intervals among studies assessing the psychometric properties of the scale. [4] The formula used to calculate the sample size [10] was *n = 16p(1-p)/w*^*2*^, where *p* was the lowest expected ICC (0.85) and *w* was the maximum reported width (0.15) of the 95% confidence interval. [4] The sample size was calculated to be 91; however 215 individuals were prospectively recruited. We enrolled 150 male ACL patients (27.1±7.0 years) for the following reasons: i) a minimum number of 100 subjects is required to ensure stability of the variance-covariance matrix in dimensionality analysis, [8] and ii) accounting for non-attendances at rehabilitation sessions to ensure that reproducibility testing would be done in “stable” patients. [8] Moreover, we enrolled 20 healthy (19.4±1.5 years) and 45 “at risk” (soccer players) for an ACL injury individuals (22.7±3.6 years) to evaluate interpretability of the scale. [8] These participants were recruited through direct contact during their training sessions at their sport clubs (during recruitment 3 potential candidates for the healthy and 8 potential candidates for the “at risk” group declined to participate).

### Inclusion and exclusion criteria

All participants had to be at least 18 years of age and willing to provide written informed consent. Patients were eligible for the study if they had an ACL injury and either had a reconstruction or were undergoing conservative care. Participants were excluded if they had other orthopaedic conditions (e.g. low back pain, referred spinal symptoms, immobilised fracture) that would significantly compromise their physical functional status. For the healthy and “at-risk” groups, additional exclusion criteria were pain and functional deficits of the lower limbs during sports participation. All asymptomatic individuals who met these criteria and assessed by a physiotherapist were included.

### Procedures, measurement and psychometric properties

The LEFS-MSAr was administered to all participants (n = 215) and completed twice within 3.3±2.0 days in the presence of one of the investigators. On completion of the questionnaire if the investigator identified a missing item the patient was questioned why. If it was an oversight, the patient was asked to respond to the item. Where the item was as not-applicable an explanation was requested and recorded. This interval between test-retest was chosen as the time period short enough to guarantee that clinical change has not occurred. [5, 8] Furthermore, based on previously published methodology, [11, 12] to ensure stability of the condition we only included participants who self-rated their condition as unchanged at the second assessment occasion.

The assessed measurement properties of the LEFS-MSAr questionnaire are presented in Table 2.

**Table 2:** Measurement and psychometric properties assessed for the LEFS-MSAr questionnaire.

Validity Testing	
Face validity [13–15]	Face validity of LEFS-MSAr was assessed: a) in 3 steps of the translation/cross-cultural adaptation process (validation of translation, review and finalization, and pretesting), b) during the content analysis procedure (see content validity), c) by the participants that appraised the extent to which the instrument assessed their condition after completion of the questionnaire, and d) by the authors.
Content validity [13, 15]	Content validity was tested in all 20 items of LEFS-MSAr through a structured content analytic method. [16] The items were distributed to 10 judges (native Arabic-speaking patients) during the fifth step of translation and another 8 judges (1 academic, 2 sport physicians, 2 physiotherapists, and 3 professional athletes; all holding higher degrees in relevant areas) after the pretesting process. The judges matched each of the 20 items based on their content to a five-point Likert scale (1 = poor, 2 = fair, 3 = good, 4 = very good, or 5 = excellent match). This quantified item ratings allowing evaluation of judgments using quantitative statistical procedures. Finally, the content validity was also assessed by the 20 patients participated in the pre-testing phase of cross-cultural adaptation.
Construct validity [13–15]	Construct validity was evaluated by convergent validity. Concerning LEFS, a criterion scale does not exist; instead, the International Knee Documentation Committee subjective knee form (IKDC) [17] was the only region-related scale available for Arabic-speaking patients (translated and adapted, not published) for which we expected a high correlation (>0.80) with the LEFS based on a previous cross-cultural adaptation [18] that enrolled patients with a variety of knee disorders. Patients with ACL injury (n = 50, 28.6±8.4 years) during the initial assessment and before the administration of LEFS-MSAr were asked to complete the IKDC questionnaire.
Structural validity [13, 15]	Following guidelines [8] and given that the unidimensionality of LEFS has been confirmed, [1, 4] the structural validity as part of construct validity of LEFS was examined by using firstly confirmatory factor analysis (CFA). A poor model fit would be followed by an exploratory factor analysis (EFA) in order to establish its dimensionality and whether the items in the questionnaire group together in a consistent and scrutable way.
Known groups validity [13, 15]	Hypothesis-testing as part of construct validity evaluation was conducted by known groups validity and using two approaches: a) the contrasted-groups approach was tested by sampling the LEFS-MSAr mean scores of three distinct groups (early, intermediate and advanced stage of ACL rehabilitation) known to be high, mid, and low in the construct being measured [5] (n = 150), and b) by comparing the total LEFS score of the participants’ groups. The healthy/“at risk”, and patient group are distinct subgroups that are estimated to score differently in the LEFS (n = 215). We expected a higher score at healthy and “at risk” groups compared to the patient group.
**Reliability Testing**	
Inter-item reliability [13, 15]	The inter-item reliability of the LEFS was assessed by using Cronbach’s alpha coefficient (α) that is considered to be the optimal estimate of the internal consistency and structural validity of the instrument where the scale is unidimensional. [15]
Test-retest reliability [8, 13–15]	To evaluate test-retest reliability the scale was administered to all the participants (n = 215) twice within 3.3±2.0 days. The time interval between repeated administrations should be long enough that the respondents do not recall their original responses, but short enough to ensure clinical stability of the condition. We re-tested only patients that self-rated their condition as unchanged between administrations.
Longitudinal reproducibility [19, 20]	A measuring instrument should be reproducible while assessing changes in a given condition (longitudinal reproducibility). [19] In the present study we repeated the test-retest procedure (range 3 to 5 days) in a group of ACL patients (n = 20) after 8 weeks from initial administration of the LEFS-MSAr.
**Utility evaluation**	
Feasibility and acceptability [13, 14]	To appraise the acceptability and the ease of administration of the LEFS-MSAr we recorded the time spent by the participants filling it out and the percentage of unanswered questions.
**Other properties**	
Responsiveness [8, 20, 21]	Responsiveness reflects the ability of a questionnaire to detect clinically important changes over time or the validity of the change score. The receiver-operating-characteristic (ROC) curve analysis was used to determine the LEFS-MSAr change scores that best discriminate between the patients who were reported to achieve their goals and change group of rehabilitation (anchor) and the patients who did not change group.
Interpretability [8, 15]	Interpretability is defined as the degree to which one can assign qualitative meaning to an instrument’s quantitative scores or change in scores. [8, 15] To aid in interpreting scores on LEFS-MSAr: a) normative scores of healthy and “at risk” for an ACL injury individuals were presented, b) the population of post-operative and conservatively managed patients was divided into three groups (early, intermediate, and advanced) by the treating physiotherapists using strict functional objective criteria (S1 Table); individual scores were presented and compared to their group/functional status, and c) we compared the scores of patients between two time points following treatment of known efficacy. It was expected based on published data [1, 5] that the LEFS scores would increase with days since surgery or injury, since one can reasonably expect patients to recover over time. We expected at least 9-point increase for a true difference according to previously published data from ACL patients. [5] More specifically, distribution-based methods were used to examine the magnitude of the change in the LEFS-MSAr and not the validity of the change score. The LEFS-MSAr was administered to a group of ACL patients (n = 90; 26±6.2 years) on two occasions 2 months apart. We hypothesized that clinically meaningful differences should be displayed in scores (higher scores) obtained by these patients as a result of rehabilitation. Minimal clinically important difference should not be considered a fixed property of the instrument; but we added this property in our analyses in order to interpret the change of scores over time in that specific population. [8, 20] Several strategies have been proposed for relating instruments’ scores to independent standards that are themselves interpretable and serve as anchors, such as patient-rated global change or functional measures. [20] We calculated the Guyatt responsiveness index [19], as well as we implemented four anchor-based methods: a) the comparison of change scores with the Minimal detectable change (MDC_95_) as was calculated from the LEFS-MSAr scores that represents the true change beyond the error of the measurement, b) we compared the change in total scores with the smallest real difference (SRD) as was calculated from the mean change score of the ‘stable’ patients at 2-month follow-up (patients that did not change rehabilitation group). [22]
Ceiling and floor effects [8]	The LEFS-MSAr would be considered to have ceiling and floor effects if more than 15% of the patients scored the maximum and minimum possible score respectively. Relative to each item of the questionnaire ceiling and floor effects were considered to have occurred if at least 75% of the patients scored the maximum or minimum score to that item, respectively.

### Statistical analyses

Statistical analyses were performed using SPSS v19.0 and AMOS v24.0. The level of significance was set at p<0.05. Descriptive statistics were used to calculate the characteristics of the participants, the scores of LEFS-MSAr and International Knee Documentation Committee subjective knee form (IKDC) questionnaires, the mean of days between test-retest, the mean scores for each item for comparability of language and similarity of interpretability, acceptability, and the ceiling and floor effects. Missing values were listwise excluded.

#### Validity testing

For item-content relevant analyses the judges’ ratings were evaluated based on the validation procedure of Aiken’s item-content validity coefficient (*V*). [23] The *V* statistic provides statistical significance of judges’ ratings about an item’s content-match with its construct and its values range from 0 to 1 (1 = perfect agreement). The values were then compared against a right-tailed binominal probability table provided by Aiken [23] (*V* scores >0.70 considered as having acceptable validity, p<0.01). Convergent validity was assessed with Spearman *rho* (*r*) between the scores obtained from LEFS-MSAr and IKDC subjective knee form. [17]

A confirmatory factor analysis (CFA) on the LEFS items was conducted to evaluate the fit of the data to the hypothesised one-factor model. [1] The model parameters were estimated using the maximum likelihood method. The model was assessed by the standardized route mean square residual (SRMR), the root mean square error of approximation (RMSEA) (values <0.05 = good, <0.08 = adequate, >0.08 = poor fit for both indices), the comparative fit index (CFI) and the Tucker Lewis index (TLI) (values >0.95 = good, >0.90 = adequate, <0.90 = poor fit for both indices). [24] To explore the factorial validity of LEFS an exploratory factor analysis (EFA) (principal axis factoring) with varimax rotation was used. Eigenvalues over 1 were chosen and extracted, and items loading more than 0.40 were regarded as loading on a specific factor. Items loading more than 0.40 on 2 factors were assigned to the factor with a higher correlation. [25]

Known groups validity and group differences were calculated using the Kruskal-Wallis test, with post hoc comparisons using the Mann-Whitney *U*-test with Bonferroni correction for multiple testing, resulting from the formula *k(k– 1)/2*, where *k* is the number of groups (p_adj_ = 0.017).

#### Reliability testing

The interrelatedness among the items of the LEFS was assessed by using Cronbach’s *α*. Values of 0.70–0.90 have been proposed as a measure of good internal consistency. [8] Reproducibility was evaluated by using both Spearman’s *rho* and 2-way random effects model Intraclass Correlation Coefficient, type agreement (ICC_2,1_), because systematic differences are considered to be part of the measurement error. [8, 26] As a measure of agreement the absolute measurement error was expressed as the standard error of measurement (SEM_Agreement_ = SD x √1-ICC), including the systematic differences in order to distinguish them from real changes, e.g., due to treatment. [8] In addition, the minimal detectable change (MDC_95_ = 1.96 x √2 x SEM) was calculated, which corresponds to the minimal within-person change in score that, with p<0.05, can be translated as a real change above measurement error. [8, 27] Bland-Altman methods were used to indicate absolute agreement for test–retest measurements including a scatter plot of differences between applications, with 95% limits of agreement (mean change in scores of repeated administrations). [28]

#### Responsiveness and interpretability

The Wilcoxon test, using scores separated by 2 months was conducted to add in instrument’s interpretability. Also, effect size (ES) by using both baseline and pooled standard deviation (SD) and standardised response mean (SRM) were calculated [20] and interpreted according to published recommendations (values of 0.20, 0.50, and 0.80 or greater represent small, moderate and large responsiveness, respectively). [29]

The Guyatt responsiveness index (GRI) was calculated as the ratio of the average change in improved patients divided by the SD of the change in stable patients as indicated by the rehabilitation stage. If the GRI was larger than 1, we considered the magnitude of change as acceptable. [19] Additionally, we estimated the magnitude of change (minimal clinically important difference—MCID) that is considered important to distinguish groups and to evaluate change over time by comparison of change scores with MDC_95_, smallest real difference (SRD), The receiver-operating-characteristic curve (ROC), and area under the curve (AUC). Accordingly, to add in the interpretability of LEFS-MSAr we calculated the proportion of patients with a change score equal to or larger than the MDC_95_ for improved patients, or based on the SRD calculated (SRD = 1.96*SD_change_) [8] from the patients that were stable at the follow-up (according to rehabilitation groups’ allocation–S1 Table).

The ROC curve was also used to provide the true-positive rate (sensitivity) versus the false-positive rate (1 –specificity). The most upper left point in the diagram represents the optimal cut-off change score, which most effectively discriminates between patients who have improved and those whose condition is unchanged. [22, 30] Additionally, the AUC reflects the probability of correctly discriminating between improved and non-improved patients. This area varies from 0.5 (the questionnaire does not discriminate more effectively than chance) to 1.0 (perfect discrimination). [22, 31]

## Results

### Translation and cross-cultural adaptation

During the steps of harmonization and validation of translation the native Arabic members of the committee and individuals used for formal evaluation and cognitive debriefing revealed a potential for 5 activities that were not entirely relevant to the Arabic culture and required modification. At this point, in order to achieve cultural content equivalence, experts in physiotherapy that have an intimate knowledge of the cultural habits across the Middle East North Africa region were also consulted. Discussions were held, and a consensus formulated that 5 items would be changed (Table 3). Following these changes, the steps 4, 5 and 6 of the translation process (Table 1) were repeatedly conducted as required. These changes were distributed to the original authors of the LEFS [1] as well as other authors who have published validation studies of the North American version of the LEFS, [32] and the changes were found to be acceptable (Alcock K, personal communication 2013).

**Table 3:** Items of the LEFS modified to suit for Arabic culture.

Item	Original Item (from North American Culture)	Modified item suitable for Arab Culture
**c**	Getting into or out of the bath	Arabic sitting on the floor
**e**	Putting on your shoes and socks	Kneeling on the floor to pray
**k**	Walking 2 blocks	Walking 200 meters
**l**	Walking a mile	Walking 1.5 kilometers
**o**	Sitting for 1 hour	Sitting in a chair for 1 hour

One change was required in the instructions for the patient using the LEFS to clarify the meaning in Arabic of “do you or would you have any difficulty”. Consensus was reached among the committee and a small change was made to the instruction. The other two translation issues were not as easily resolved. Item “c” asks about Arabic sitting on the floor and item “f” asks about squatting. Both activities do not have a sufficiently detailed single word or phrase in Arabic that is consistent across all Arabic speaking countries or in modern standard Arabic. Additionally, when the tool was presented for validation of translation, these 2 questions were consistently highlighted as unclear in what was being asked. Consequently, the decision was made to partially abandon a literary description in favour of an image that clearly demonstrates the activity referred to in the question. Distribution of the LEFS-MSAr with the images was unanimously preferred among all the participants from the target population with no other changes required.

### Validity testing

An overview of the measurement properties of both the LEFS-MSAr and the original version are presented in Table 4.

**Table 4:** Summary of measurement properties of the original LEFS and LEFS-SMAr questionnaire.

Measurement property	LEFS-SMAr	LEFS-original*
**Convergent validityϮ**		
**ACL patients–LEFS first assessment (n = 50)**	**ρ = 0.85, p<0.001**	**r = 0.80**
**ACL patients–LEFS second assessment (n = 50)**	**ρ = 0.88, p<0.001**	
**Factorial validity**		
**Factor structure**	**2-factor solution**	**1-factor solution**
**Variance explained**	**78.145%**	
**Communalities**	**0.695–0.906**	
**Factor loadings**	**0.58–0.85**	**0.58–0.86**
**Internal consistency**		
**Cronbach’s α (n = 150)**	**0.965**	**0.96**
**Cronbach’s α ADL**	**0.982**	
**Cronbach’s α strenuous**	**0.934**	
**activities**		
**Test-retest reliability**		
**ICC (n = 215)**	**0.983 (95%CI: 0.977–0.987)**	**0.94 (95%CI lower 0.89)**
**ICC ACL (n = 150)**	**0.976 (95%CI: 0.964–0.983)**	
**ICC “at risk” (n = 45)**	**0.869 (95%CI: 0.772–0.926)**	
**ICC healthy (n = 20)**	**0.803 (95%CI: 0.685–0.921)**	
**Test-retest reliability**		
**Spearman’s rho ACL**	**ρ = 0.978, p<0.001**	**Not reported**
**Spearman’s rho “at risk”**	**ρ = 0.840, p<0.001**	
**Spearman’s rho healthy**	**ρ = 0.918, p<0.001**	
**Longitudinal reproducibility**		
**ICC ACL (n = 20)**	**0.988 (95%CI: 0.969–0.995)**	**Not reported**
**Agreement**		
**SEM ACL**	**3.34**	**3.9**
**SEM “at risk”**	**0.64**	
**SEM healthy**	**1.25**	
**Minimal Detectable Change**		
**MDC95(%) ACL**	**9.26 (11.57)**	**9.0 (MDC90)**
**MDC95(%) “at risk”**	**1.77 (2.21)**	
**MDC95(%) healthy**	**3.46 (4.32)**	
**Interpretability**		
**ES using baseline SD**	**0.71(Glass’ Δ)**	**Not reported**
**ES using pooled SD**	**0.81(Cohen’s d)**	**Not reported**
**SRM**	**1.17**	**Not reported**
**GRI**	**2.2**	**Not reported**
**SRD**	**16.1**	**Not reported**
**MCID**	**9 points**	**Not reported**
**Responsiveness**		
**AUC (95%CI)**	**0.781 (0.676 to 0.886)**	**Not reported**

*The original LEFS was tested in general lower extremity musculoskeletal conditions.

^Ϯ^Convergent validity was assessed using IKDC as comparator in the Arabic version, while in the original version the SF-36 was used.

Abbreviations: LEFS, lower extremity functional scale; ACL, anterior cruciate ligament injury; ADL, Activities of daily living; ICC, intraclass correlation coefficient; SEM, standard error of measurement; ES, effect size; SD, standard deviation; SRM, standardized response mean; GRI, Guyatt responsiveness index; SRD, smallest real difference; AUC, area under the curve; MCID, minimal clinically important difference.

The face validity of the questionnaire was rated as excellent from participants at pre-testing, expert committees, judges at item content analyses, ACL injury patients, and authors. Content validity was assessed through a structured content analytic method [16] and characterised as being well addressed by all 10 judges and 20 patients at pre-testing. All 20 items presented *V* values ranging from 0.725 to 1.0 (p<0.01). Convergent validity was demonstrated by a high association between the scores of LEFS-MSAr and IKDC Subjective Knee Form [17] as expected (both test and retest rho>0.80).

#### Structural validity

**Fig 1:**
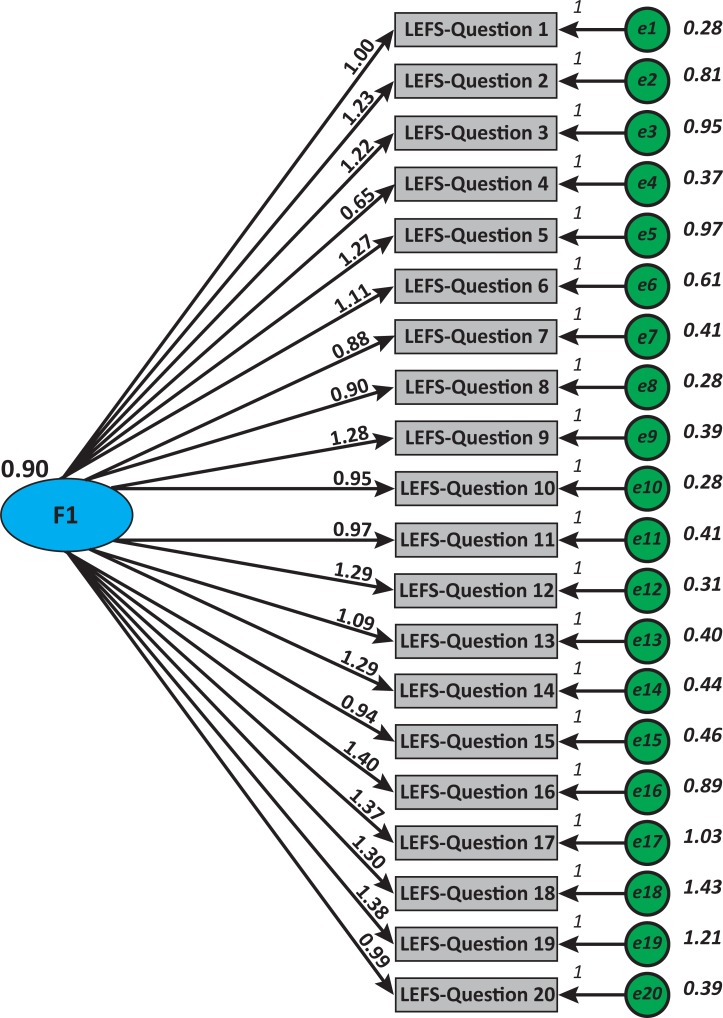
Confirmatory factor analysis. Model of the hypothesized 20-item 1-factor structure for the modern standard Arabic Lower Extremity Functional Scale (LEFS-MSAr).

**Fig 2:**
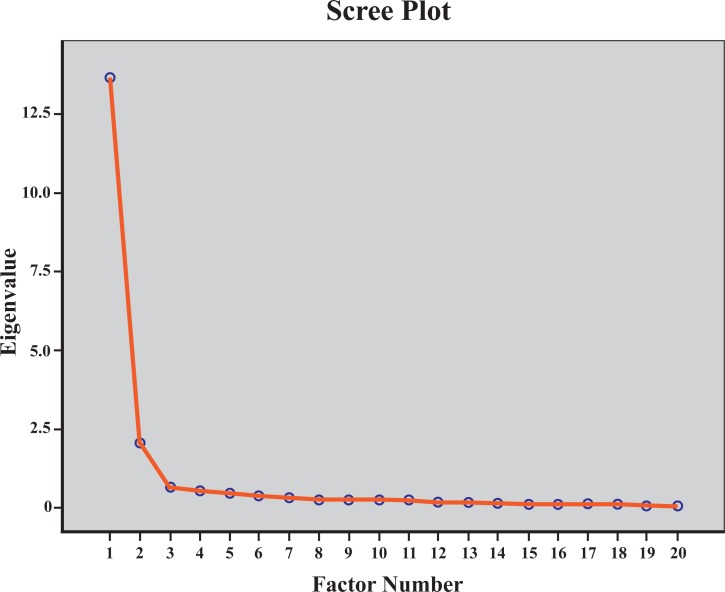
Exploratory factor analysis. Scree plot of eigenvalues form the 20-item modern standard Arabic version of Lower Extremity Functional Scale (LEFS-MSAr).

The data from 215 participants were used in the CFA. The one factor model solution exhibited poor fit (*x*^*2*^ = 1563.77; *x*^*2*^/df = 9.2; SRMR = 0.0878; RMSEA = 0.195(0.186–0.204), pclose<0.001; CFI = 0.744; TLI = 0.714). CFA suggested that more than one factor underlie the LEFS-MSAr items in an ACL injury population (Fig 1). In the EFA analysis, the Kaiser-Meyer-Olkin measure (KMO) verified the sampling adequacy for the analysis (*KMO* = 0.956), which is well above the acceptable limit of 0.5. [33] Bartlett’s sphericity test (*x*^2^(190) = 5435.096, p<0.001) indicated that the correlations between items were sufficiently large for EFA. The analysis and the examination of the screeplot (Fig 2) indicated a 2-factor solution with eigenvalues over Kaiser’s criterion of 1 explained 78.14% of the total variance (Table 5). The items that cluster on the same components suggested that component 1 represents relatively light activities of daily living and component 2 sport related and strenuous activities for the knee. For that reason, we calculated internal consistency coefficient for each unidimensional subscale separately (Table 4).

**Table 5:** Exploratory factor analysis with varimax rotation suggesting a 2-factor solution.

	Rotated factor loadings	
Item	Activities of daily living	Sports and strenuous activities
h. Performing light activities around your home	**0.85**	0.31
k. Walking 200 meters	**0.84**	0.26
g. Lifting an object, like a bag of groceries from the floor	**0.82**	0.26
j. Getting into or out of a car	**0.81**	0.38
a. Any of your usual work, housework or school activities	**0.80**	0.39
t. Rolling over in bed	**0.78**	0.36
d. Walking between rooms	**0.76**	0.19
o. Sitting in a chair for 1 hour	**0.74**	0.34
m. Going up or down 10 stairs (about 1 flight of stairs)	**0.74**	0.43
k. Walking 1.5 kilometres	**0.71**	0.55
f. Squatting	**0.68**	0.44
i. Performing heavy activities around your home	**0.67**	0.57
n. Standing for 1 hour	**0.67**	0.55
r. Making sharp turns while running fast	0.20	**0.92**
q. Running on uneven ground	0.30	**0.90**
s. Hopping	0.29	**0.86**
p. Running on even ground	0.39	**0.84**
b. Your usual hobbies, recreational or sporting activities	0.42	**0.76**
e. Kneeling on the floor to pray	0.44	**0.70**
c. Arabic sitting on the floor	0.52	**0.58**
Eigenvalues	13.64	1.97
% of variance	68.27	9.87
Total Variance %	**78.145**	

#### Known group validity

Regarding normative values, Kruskal-Wallis tests revealed significant differences (p<0.001) for mean LEFS-MSAr scores at first and second administration. No within group differences were found at both administrations. ACL injured patients scored significantly lower (p<0.017) than both athletes “at risk” and healthy individuals (Table 6).

**Table 6:** Total scores of the LEFS-SMAr questionnaire in the groups of participants.

Group	N	Test*	Re-test*
ACL	150	51.0 ± 21.2 (47.6–54.4)	52.4 ± 21.9 (48.9–55.9)
At Risk	45	78.6 ± 2.2 (78.0–79.3)	78.9 ± 2.0 (78.4–79.6)
Healthy active	20	78.3 ± 2.2 (77.3–79.3)	77.4 ± 3.4 (75.8–79.0)

*Data are presented as mean ± SD (95% CI)

The Mann-Whitney test did not reveal significant differences between healthy and at risk groups at both assessments (U_test_ = 400.0, U_retest_ = 307.5, p>0.017 respectively), but ACL injury patients scored significantly lower at both assessments than at risk (U_test_ = 244.0, U_retest_ = 307.5, p<0.001 respectively) and healthy group (U_test_ = 130.5, U_retest_ = 264.5.5, p<0.001 respectively).

Abbreviations: LEFS-SMAr, lower extremity functional scale Arabic version; N, sample size; ACL, anterior cruciate ligament injury patients; healthy active, healthy active individuals.

**Fig 3:**
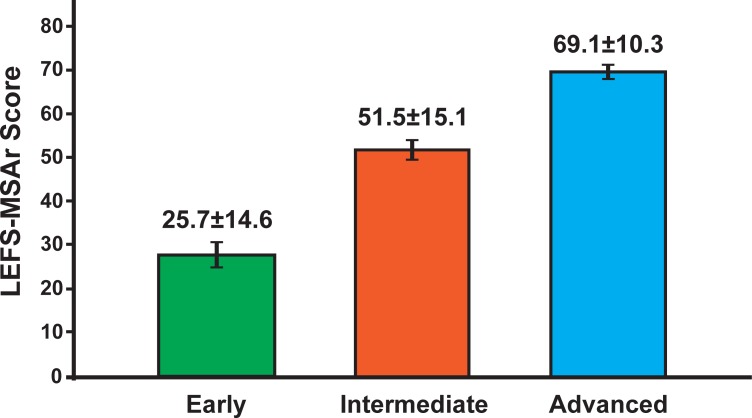
LEFS-MSAr scores according to the ACL rehabilitation stage. LEFS-MSAr mean(±SD) for each of the ACL in terms of rehabilitation stage groups. Mean total score and standard error values are depicted in the graph. Significant differences were found between scores for early (median = 25.0) and intermediate (median = 53.0) group (U = 264.500, p<0.001), early and advanced (median = 72.0) group (U = 24.000, p<0.001), and intermediate and advanced group (U = 456.000, p<0.001). Abbreviations: LEFS-MSAr, lower extremity functional scale modern standard Arabic version; N, sample size; ACL, anterior cruciate ligament injury patients.

For contrasted-groups validity testing, Kruskal-Wallis test showed significant differences (*x*^2^(2) = 91.012, p<0.001) for the LEFS-MSAr scores with respect to the ACL rehabilitation stage, with a median score of 25.0 for “early”, 53.0 for “intermediate”, and 72.0 for “advanced” (Fig 3).

#### Reliability testing

Reliability results are presented in Table 4. The Cronbach’s alpha for internal consistency was 0.965, and the Cronbach alpha if item deleted (for each item) varied from 0.962 to 0.965. The ICC_2,1_ total was 0.98 with the 95% confidence interval (CI) of 0.98 to 0.99. The SEM was calculated to be 3.34 points and the MDC_95_ 9.26 points for the ACL injured patients. LEFS-MSAr was also proven longitudinally reproducible presenting an ICC_2,1_ of 0.99, a SEM of 1.42 points and a MDC_95_ of 3.94 points.

**Fig 4:**
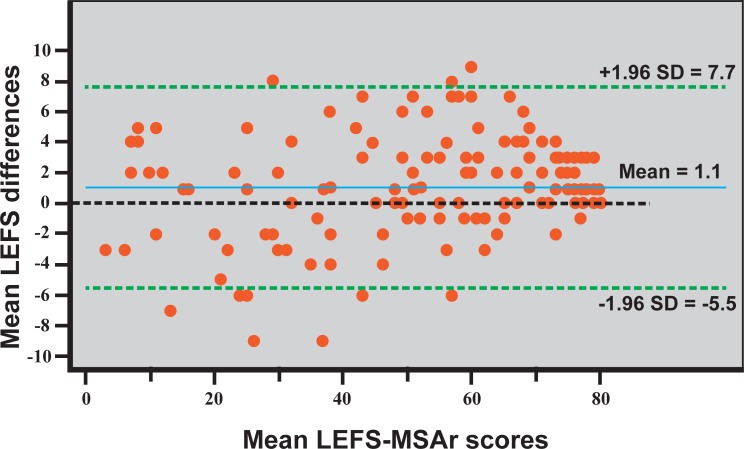
Bland-Altman plot. A Bland-Altman plot visualizing the agreement for test-retest, with the limits marked as maen±SD difference. Means and differences were calculated using total original scores of the scale. Abbreviations: LEFS-MSAr, lower extremity functional scale modern standard Arabic version.

A Bland-Altman plot (Fig 4) showed that the differences between two assessments were plotted around the zero line and within the limits of agreement (-5.5 to 7.7) with a few outliers. Moreover, the zero line was within the 95% CI of the mean difference indicating no systematic bias.

### Utility evaluation

The completion of the questionnaire required 3 to 5 minutes maximum and none of the participants reported language comprehension or semantic problems. All participants completed the full LEFS-MSAr, resulting in the maximum response rate.

### Other properties

#### Responsiveness

**Fig 5:**
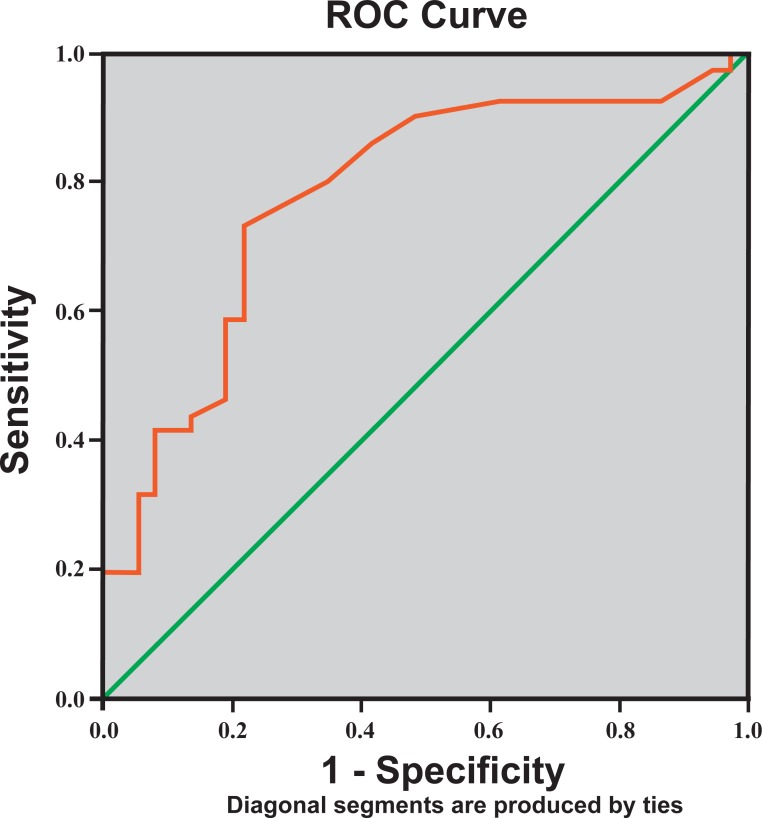
Receiver-operating-characteristic (ROC) curve. Receiver-operating-characteristic (ROC) curve illustrating the relationship between sensitivity and complement of specificity (1-specificity) for the modern standard Arabic version of Lower Extremity Functional Scale.

The ROC analysis indicated that LEFS-MSAr (Fig 5) moderately discriminated improved and non-improved patients with an AUC associated with this value of 0.781 (95%CI 0.676 to 0.886). The represented sensitivity and specificity were 0.85 of 0.65, respectively.

#### Interpretability

The LEFS-MSAr was administered to a group of ACL patients (n = 90) on two occasions two months apart. Twelve patients were excluded due to subsequent ACL reconstructive surgery.

The Wilcoxon test revealed statistically significant changes of the LEFS-MSAr from first (Median = 50.5) to second (Median = 67.5) administration for this group (Z = -7.62, p<0.0001) representing large effect sizes (Table 4).

Based on a change score equal or larger to the MDC_95_ at the re-administration of the LEFS-MSAr, 59.0% of the patients were rated as improved, while 41% were found with no change. However, at the final assessment 38 ACL patients were at the advanced (final) stage of rehabilitation where little further improvement is expected.

Based on change of rehabilitation group (early, intermediate, and advanced) 53.1% (mean_change_±SD_change_ = 18.1±12.6) of the patients were rated as improved, while 46.9% (mean_change_±SD_change_ = 7.5±8.2) were found with no change.

The SRD calculated from the change scores for the ‘stable’ patients was 16.1 points, indicating that an individual had to change at least 16 points on the LEFS-MSAr to be judged as having really changed. Based on SRD the sensitivity to change of the LEFS-MSAr was 36%. Seven of the stable patients (11%) had a change score higher than SRD, indicating a specificity to change of around 89%.

**Fig 6:**
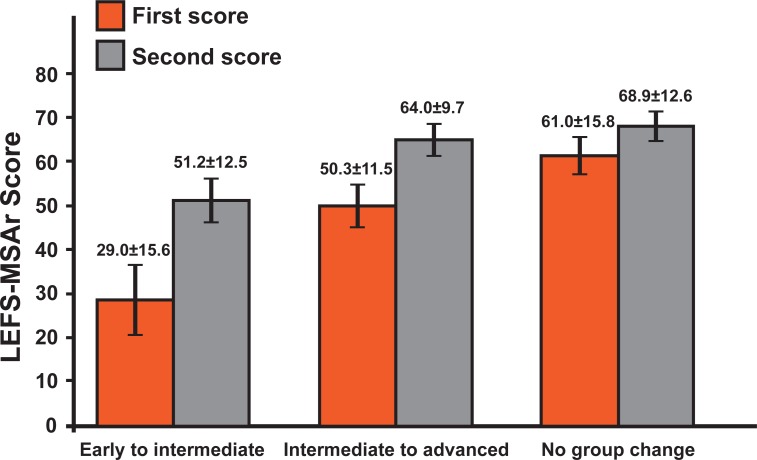
Mean LEFS-MSAr scores over 2 months. Mean LEFS-MSAr scores over a two-month test-retest, grouped by independently rated ACL rehabilitation group. Abbreviations: LEFS-MSAr, lower extremity functional scale modern standard Arabic version.

Normative scores of LEFS-MSAr are presented in Table 6, while individual patient scores according to functional status are presented in Fig 3. Significant changes over time were found for LEFS-MSAr (p<0.0001) with a large ES following rehabilitation. Fig 6 documents the two-month test-retest data with patients classified according to their independently rated rehabilitation status. The mean change in LEFS-MSAr score was significant (p<0.01) for those subjects who changed groups (22 and 14 points, respectively), and exceeded the documented MDC (9 points), whereas the change for those subjects who stayed in the same group was non-significant (8 points).

#### Ceiling and floor effects

No floor effect was found for LEFS-MSAr total score at first (0%) or second assessment (0%), nor a ceiling effect (1.3% and 2.6%, respectively). Additionally, no individual items of the scale were scored at their maximum or minimum score by more than 75% of the patients at first (floor range 6–44.4%, ceiling range 6–64.2%) or second assessment (floor range 3.3–49%, ceiling range 13.9–74.2%).

## Discussion

The LEFS was translated into modern standard Arabic, significant cultural adaptations were implemented, and the LEFS-MSAr demonstrated excellent psychometric properties as hypothesised in a cohort of patients with ACL injury.

### Translation and cross-cultural adaptation

There are at least 12 major sets of guidelines available for questionnaires translation [7] and to our knowledge there is no consensus on a set of rigid procedures in the area of translation and cross-cultural adaptation. To overcome this problem and ensure translation quality we synthesised a rigorous process from published recommendations. [2, 6, 7]

A previous translation of the LEFS into Arabic [3], presented some difficulties in cultural relevance and comprehension. [6] Specifically, administering this version to our multinational Arabic-speaking patients we realized that patients were not able to understand some questions, as for example item “f” inquiring about squatting. Moreover, we observed that the item “c” was translated as “walking in and out of the bathroom” instead of “getting into or out of the bath”. Finally, some items were culturally irrelevant and had to be rephrased to reflect modern Arabic reality. Examples of problems the authors have encountered may be illustrative: i) item “o” inquiring about sitting does not reflect the Arabic culture of sitting (Arabic sitting on the ground), and ii) item “e” inquiring about “putting on shoes and socks” may not reflect an activity of older individuals that usually wear sandals. Accordingly, we cross-culturally adapted the LEFS into modern standard Arabic aiming to produce in a widely comprehensible and practical tool for use in Arabic culture across the Middle East North Africa region. Given that there are potential cultural differences in the interpretation of many terms, appropriate attention was given to cultural nuances by using bicultural members in the committees and implementing a formal translation validation by using native Arabic-speaking patients representing the Middle East North Africa region population.

### Validity testing

As hypothesised, the LEFS-MSAr demonstrated good translational and construct validity. Evaluation of content validity by a structured analytic method [16] added to the psychometric properties of LEFS, as to our knowledge this is reported first time, [4] since most of previous studies used floor and ceiling effects to examine this form of validity. [4] The results also confirmed our hypothesis regarding the convergent validity of LEFS-MSAr with the IKDC Subjective Knee Form [17] scores presenting a high positive correlation as previously reported. [18]

Strength of the present study was the structural validity evaluation by initially using CFA followed by EFA. However, the CFA/EFA revealed a 2-factor structure that did not support the hypothesized unidimensionality as previously reported. [1, 3, 34–37] The unidimensionality of LEFS has been questioned by another study used CFA [38] and the factor complexity of LEFS has been demonstrated before exhibiting 3 factors in patients with hip and knee osteoarthritis, [39] and 2 factors in patients with total hip and knee replacement [40] and general musculoskeletal disorders of the lower extremity. [41] The configuration of patient population and conditions involved may explain these discrepancies.

### Reliability testing

An excellent test-retest reliability was demonstrated for ACL injured patients (ICC = 0.976) within the range (0.85–0.998) [4] of reported reproducibility in multiple conditions among studies. Also, we assessed the longitudinal reproducibility of LEFS-MSAr by repeating the test-retest procedure after 8 weeks from initial administration; a property that is almost completely neglected in the evaluation of instruments. [20] Again the LEFS-MSAr showed excellent temporal stability presenting an ICC of 0.988.

Regarding absolute reliability, the SEM calculated for our ACL injured patients scores was 3.34 points which is within the reported range in the literature (0.88–5.6), [4] and comparable to the SEM reported for ACL reconstructed patients (3.7) [5] and patients with various knee disorders (3.6). [18]

### Interpretability and responsiveness

Clinically meaningful score differences with large effect sizes, an SRM of 1.17 and a GRI of 2.2 were displayed, reflecting ability of LEFS-MSAr to effectively distinguish changes over time. The ES reported herein (0.71-Glass’ Δ and 0.81 Cohen’s d) is far less than that in other studies [42–45] ranging from 1.26 to 3.33. [4] ES is strongly affected by the type of injury (i.e. acute or chronic) and the interval between test-retest and must be interpreted by clinicians with caution. A big enough interval between two evaluations, as for example in the study of Lin et al, [43] (6 months) and a sample configuration of patients with non-chronic conditions (>65%) combined with long evaluation interval as in Cruz-Diaz et al, [44] may lead in ES overestimation (3.33 and 2.3, respectively).

MCID should not be considered a fixed property of the instrument, nor responsiveness as a constant characteristic of a measure, [8, 20, 46] while different methods of MCID calculation can result in different values of MCID, particularly when baseline scores are characterized by different levels of severity. [47] In our ACL population LEFS-MSAr was found sensitive to change with an MCID of 9 points marginally not meeting the requirement of being greater than the MDC_95_ (9.26 points). Despite that, a change of greater than 9 points can make the clinician reasonably confident that this change is not due to error, but also is a clinically meaningful change. [1] In the majority of studies assessing psychometric properties of LEFS [1, 41, 48, 49] the MCID did not exceed the measurement error. Calculation of indices like MDC is dependent on the selected CI range with related *z* value and following the COSMIN recommendations [8] the MDC corresponding to 95% CIs is preferred. Taking this into account, not even in the original publication did the reported MCID exceed the MDC_95_ (10.8 points); these results indicate that further investigation into these properties in patients with lower limb conditions is necessary.

### Limitations

The study sample is specific to ACL injured patients of male gender. The extent to which our results can be generalized to female or patients with other conditions is unknown and has to be elucidated in future studies. Also, our methodology considered only classical test theory; given the inconsistency in available literature regarding the unidimensionality of the scale a rigorous Rasch analysis is much needed to examine in detail the internal structure of the LEFS. Given that the assumption of unidimensionality was not met, the reported Cronbach’s alpha may have overestimated the internal consistency of the scale. Finally, a possible deficiency of the study is the usage of achievement of functional treatment goals in MCID evaluation compared to all previous studies that used global change rating scale.

## Supporting information

S1 File Modern standard Arabic version of LEFS (LEFS-MSAr). (PDF)

S2 File Anonymized data. (XLSX)

S1 Table ACL rehabilitation progression criteria. (DOCX)
