# Supplementary material for: Translation into modern standard Arabic, cross-cultural adaptation and psychometric properties’ evaluation of the Lower Extremity Functional Scale (LEFS) in Arabic-speaking athletes with Anterior Cruciate Ligament (ACL) injury
Source: PLoS One. 2019 Jun 10;14(6):e0217791. doi: 10.1371/journal.pone.0217791 (PMC6557503; doi:10.1371/journal.pone.0217791)
Supplement: S1 File — (PDF) [file pone.0217791.s001.pdf]

ASPETAR سبيتار

Lower Extremity Functional Scale - Modern Standard Arabic
(LEFS-MSArab)

مقياس الأداء الوظيفي للأطراف السفلية

The **Lower Extremity Functional Scale (LEFS)** is subject to copyright, but it's free to be used for clinical or research purposes. The **ASPETAR version (LEFS-MSArab)** followed cross-cultural adaptation guidelines and was assessed for "comparability of language" and "similarity of interpretability".

Original publication: "Binkley JM, Stratford PW, Lott SA, Riddle DL. The Lower Extremity Functional Scale (LEFS): Scale development, measurement properties, and clinical application. *Physical Therapy* 1999; 79:371-383"

For further information contact:

Vasileios Korakakis (Vasileios.Korakakis@aspetar.com) or

Noor Al Marri (Noor.AlMarri@aspetar.com) or

Michael Saretsky (Michael.Saretsky@aspetar.com)

نحن نهتم بمعرفة ما إذا كنت تعاني من أي صعوبات أثناء ممارسة الأنشطة المدرجة أدناه بسبب إصابتك في الأطراف السفلية والتي تسعى للحصول على علاج لها حاليًا .يرجى تقديم إجابة عن كل نشاط.

اليوم،هل تعاني أو تتوقع بأنك ستعاني من أي صعوبة مع:

**الأنشطة**	صعوبة شديدة أو عجز عن أداء النشاط	صعوبة كبيرة نوعًا ما	صعوبة متوسطة	صعوبة ضئيلة	لا توجد صعوبة
أ. أي من الأعمال المعتادة أو الأعمال المنزلية أو الأنشطة المدرسية.					
ب. الهوايات المعتادة أو الأنشطة الترفيهية أو الرياضية.					
ج. الجلوس على الأرض الطريقة العربيةعلى 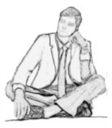					
د. المشي بين الغرف.					
ه. الركوع والسجود أثناء تأدية الصلاة.					
و. جلسة القرفصاء 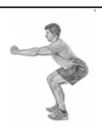					
ز. رفع أي جسم من على الأرض مثل كيس البقالة.					
ح. القيام بأنشطة خفيفة داخل المنزل.					
ط. القيام بأنشطة شاقة داخل المنزل.					
ي. الصعود إلى السيارة أو النزول منها.					
ك. المشي لمسافة 200 متر.					
ل. المشي لمسافة كيلو متر ونصف.					
م. صعود ونزول 10 درجات من السلم (حوالي مجموعة واحدة من درجات السلم).					
ن. الوقوف لمدة ساعة.					
س. الجلوس على الكرسي لمدة ساعة.					
ع. الجري على أرضية مستوية.					
ف. الجري على أرضية غير مستوية.					
ص. عمل انعطافات حادة أثناء الجري بسرعة.					
ق. الوثب.					
ر. التقلب في السرير.					
**مجموع النقاط في كل عمود**					

الدرجة:_______/80
