# Supplementary material for: Translation into modern standard Arabic, cross-cultural adaptation and psychometric properties’ evaluation of the Lower Extremity Functional Scale (LEFS) in Arabic-speaking athletes with Anterior Cruciate Ligament (ACL) injury
Source: PLoS One. 2019 Jun 10;14(6):e0217791. doi: 10.1371/journal.pone.0217791 (PMC6557503; doi:10.1371/journal.pone.0217791)
Supplement: S1 Table — (DOCX) [file pone.0217791.s003.docx]

| **Table S1** ACL rehabilitation progression criteria | |
| --- | --- |
| **Progression from early to intermediate** | **Progression from intermediate to advanced** |
| 1. No or minimal intra- or extra- articular swelling | 1. No or minimal intra- or extra- articular swelling |
| 1. Full active knee extension in supine lying | 2. Full active range of motion |
| 1. No Quadriceps lag at active straight leg raise | 3. Normal walking gait |
| 1. Complete early exercise program | 4. Isokinetic testing <30% Quadriceps difference at CON 60º |
| 1. Bilateral squat with good control and alignment (knee, pelvis, and trunk) | 5. Good knee alignment on unstable surface |
| 1. Single-leg stance for 10 seconds |  |
| 1. Almost normal/mild antalgic gait without aid |  |
